# Predictive Value of Diaphragmatic Ultrasonography for the Weaning Outcome in Mechanically Ventilated Children Aged 1–3 Years

**DOI:** 10.3389/fped.2022.840444

**Published:** 2022-03-30

**Authors:** Yelin Yao, Liming He, Weiming Chen, Hao Zhou, Guoping Lu, Jinhao Tao, SuJuan Wang

**Affiliations:** ^1^Department of Rehabilitation, Children’s Hospital of Fudan University, Shanghai, China; ^2^Pediatric Emergency Critical Care Center, Children’s Hospital of Fudan University, Shanghai, China

**Keywords:** diaphragmatic ultrasonography, weaning, children, mechanical ventilation, critical illness

## Abstract

**Background:**

There are estimated 20% of mechanically ventilated patients having difficulty in weaning from the ventilators, and the weaning process accounts for 40% of the whole ventilation time. Reliable tools are urgently needed to estimate the weaning outcome. Diaphragmatic ultrasonography, as a relatively good predictive method for the adults, was measured in this study, assessing the value of each indicator of diaphragmatic ultrasonography to predict the outcomes of ventilator weaning from mechanically ventilated children of 1–3 years old.

**Methods:**

Between November 2018 and November 2019, children who were mechanically ventilated and ready for weaning in the pediatric intensive care unit (PICU) were enrolled in the study. Diaphragmatic ultrasonography was performed to the children to measure the right diaphragm excursion (DE), contraction velocity, thickness, and diaphragm thickening fraction (DTF), which were recorded followed by spontaneous breathing trial (SBT). The receiver operator characteristic (ROC) curves were also used to assess the value of each indicator to predict the weaning outcome.

**Results:**

During this study period, a total of 72 children were enrolled, and of them, 56 children passed the weaning process, while 16 children failed. There were significant differences in DE, contraction velocity, thickness, and DTF parameters between the weaning success group and the failure group. The areas under the ROC curves (AUC) and the optimal threshold of the above indicators were as follows: 0.72 and 8.08 mm for DE, 0.71 and 26.1% for right DTF (DTF^R^), 0.71 and 20.7% for left DTF (DTF^L^), 0.78 and 14.8% for minimum DTF (DTF^MIN^), 0.79 and 26.1% for maximum DTF (DTF^MAX^), 0.71 and 1.24 mm for maximum diaphragm thickness at the end of inspiration (Dtei^MAX^), and 0.65 and 10.0 mm/s for contraction velocity.

**Conclusion:**

Diaphragmatic ultrasonography is feasible in guiding ventilator weaning, and the indicators of DE, DTF, and Dtei^MAX^ guide the weaning more accurately. Among them, DTF may act as a more reliable predictor of weaning by avoiding the influence of diaphragm development in children.

## Introduction

Invasive mechanical ventilation provides effective respiratory support for the critically ill pediatric patients and has been widely used in children in the intensive care unit (ICU). Ultimately, weaning from the ventilator is compulsory once the condition of the children with critical illness improves. The failure rate of children weaning can reach as high as 8–20%, and the failure in weaning is considered as an independent risk factor for a worse clinical outcome (1–3). Early weaning and late weaning can both lead to adverse effects on the children (3, 4). Therefore, predictive indicators for weaning among children are especially necessary. Currently, some predictive indices of weaning include the rapid-shallow-breathing index (RSBI), maximum inspiratory pressure (MIP), work of breathing, and diaphragm ultrasound. Work of breathing indices and the ultrasonic techniques have become the research hotspots of adult weaning predictive index because 75–80% of the former predictor was provided by diaphragmatic muscle, and the latter predictor could directly observe the diaphragmatic activity along with the advantages of being non-invasive and convenient. In the past 15 years of adult research, the functional indices, such as mobility, contraction speed, thickness, and thickening fraction of diaphragm ultrasound, in predicting weaning have been established (5). Among them, some functional indices have good predictive values, and there are critical values of the reference from clinical weaning (6, 7). Similarly, diaphragm ultrasound can be used for monitoring the changes of the diaphragmatic muscle function of children (8). On account of the characteristics of the growth and development of children, the diaphragmatic muscle will change as they grow. There is no relevant adjusted value of diaphragmatic muscle index in healthy children at present. So, it is relatively complex and difficult to explore the children’s functional index of diaphragmatic ultrasonography in predicting the weaning outcome. In order to reduce the bias that resulted from growth and development, a small range of children aged 1–3 years was selected for this study so as to find the appropriate predictive index of diaphragm ultrasound for weaning.

## Materials and Methods

### Participants

Pediatric patients aged 1–3 years who were mechanically ventilated for more than 48 h through endotracheal tube in the pediatric intensive care unit (PICU) of the Children’s Hospital of Fudan University between November 2018 and November 2019 were prospectively screened for inclusion. The main indications for mechanical ventilation were (1) airway protection for a patient with a decreased level of consciousness, (2) hypercapnic respiratory failure due to airway, chest wall, or respiratory muscle diseases, (3) hypoxemic respiratory failure, or (4) circulatory failure (9). The patients would be recruited if they met the following ventilator weaning criteria: (1) removal or improvement of the primary disease that led to respiratory compromise; (2) spontaneous breathing and intact central respiratory drive; (3) respiratory tract unobstructed, intact cough reflex; (4) steady hemodynamic state; (5) muscle relaxant was not used in 24 h, and dosage of sedative and analgesic drugs did not increase; (6) stable electrolyte state in the internal environment; and (7) appropriate gas exchange, positive end-expiratory pressure (PEEP) ≤ 8 cmH_2_O, and inhaled oxygen concentration (FiO_2_) ≤ 50%. The exclusion criteria were as follows: patients who had a history of diaphragm paralysis, or cervical spine injury, or neuromuscular diseases; patients who underwent thoracoabdominal surgery. All pediatric patients were weaning for their first time. This study met the requirements of the medical ethics and was approved by the Scientific and Ethics Committees of Children’s Hospital of Fudan University, approval number: (2018) 266. Informed consent from the legal guardians was acquired.

### Weaning Procedure

The subjects were assessed daily for meeting the weaning criteria. Once the patients met the weaning criteria above, diaphragm ultrasound would be performed followed by a spontaneous breathing trial (SBT). Then the patients were placed in a semi-reclining position, and an SBT was conducted with a pressure-support ventilation (PSV) mode for 2 h as others did (10). The PSV settings were pressure support at 5 cmH_2_O, PEEP 5 cmH_2_O, and FiO_2_ was the same as used before SBT. Criteria for passing SBT were as follows: respiratory rate within the predictive range (<50% increase compared with baseline), tidal volume between 5 and 7 ml/kg, SpO_2_≥ 92%, ETCO_2_ within 35–45 mmHg, heart rate within the predicted range (≤20% increase compared with baseline), and no signs of respiratory distress. Pediatric SBT failure standards were (1) clinical standards: (a) diphoresis, (b) nasal flaring, (c) increase of work in breathing, (d) tachycardia (increase of >40 times/min compared with the baseline value), (e) arrhythmia, (f) low blood pressure (according to the standard of different ages), (g) apnea; (2) laboratory standards: (a) increase of >10 mmHg of end-tidal carbon dioxide compared with the baseline value, (b) arterial blood pH < 7.32, (c) decrease of >0.07 in arterial blood pH value compared with the baseline value, (d) when FiO2 > 40%, PO2 < 60 mmHg (P/F ratio < 150 mmHg), (e) SpO_2_ decreases > 5% (3). The patients who passed the SBT would have the ventilator withdrawn immediately. Extubation and mechanical ventilation (invasive or non-invasive) was not needed within 48 h after extubation was defined as successful weaning. Failure to pass the SBT, or re-intubation, or resumption of ventilatory support within 48 h after passing the SBT was defined as failed weaning. According to the weaning outcome, the patients were divided into two groups: the success group and the failure group.

### Diaphragmatic Ultrasound Index Measurement

#### Diaphragm Excursion Measurement

**FIGURE 1:**
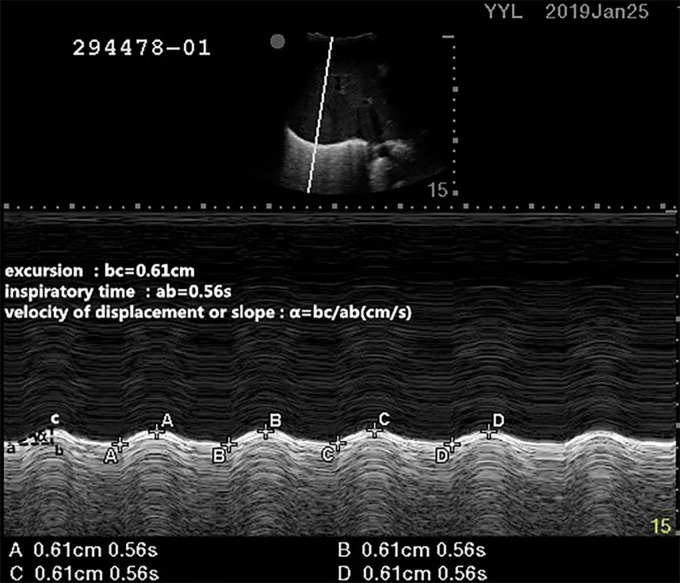
Measurements of the diaphragmatic excursion (DE) and systolic velocity by the ultrasound. During inspiration, the diaphragm contracts and moves toward the probe. During expiration, the diaphragm rebounds and moves away from the probe. The distance between them on the vertical axis during a respiratory cycle was recorded as the diaphragmatic excursion. For systolic velocity, it was equivalent to DE/time of inspiratory time.

A professionally trained physician measured the diaphragm excursion and thickness by ultrasound as previously described (11). The patients were placed in the supine position with a 30° bed head elevation. During ultrasound, pressure support ventilation was used in the recruited patients, during which a tidal volume of 6–8 ml/kg was maintained for each patient. The right side of diaphragmatic excursion was measured with a 2- to 5-MHz ultrasound probe (Edge II ultrasound system, United States). First, the B-mode was used to detect the best image of the diaphragm, and the 2- to 5-MHz ultrasonic probe was placed at the intersection of the right mid-clavicular line and costal margin to measure the DE. Taking the right hepatic lobe as an acoustic window, the probe was directed medially, cephalad, and dorsally, so that the ultrasound beam could reach perpendicularly the posterior third of the diaphragmatic muscle. Then the M-mode was used to record the DE during respiration after selecting the best image and exploration line (10). During inspiration, the diaphragm contracts and moves toward the probe. During expiration, the diaphragm rebounds and moves away from the probe. The distance between them on the vertical axis during a respiratory cycle was recorded as the DE. The average value of three consecutive respiratory cycles (since the screen could only mark eight points, it could mark up to four cycles) on the ultrasound display screen was taken to represent DE, and the systolic velocity was calculated. For systolic velocity, it was equivalent to DE/time of contraction (as shown in Figure 1) (12, 13).

#### Diaphragm Thickness Measurement

**FIGURE 2:**
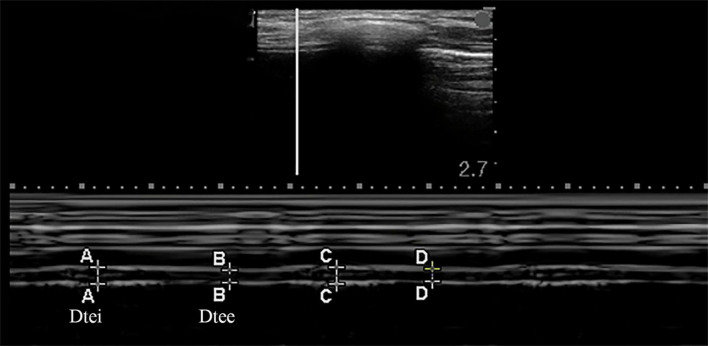
Measurements of the diaphragm thickness by the ultrasound in M-mode. Dtee, diaphragm thickness at the end of expiration; Dtei, diaphragm thickness at the end of inspiration.

The bilateral diaphragm thickness was detected by utilizing a linear array probe of frequency 6–13 MHz, which was placed in the mid-axillary line, perpendicular to the thoracic wall. The B-mode was used to display the image of the diaphragm. In this area, the diaphragm was divided into a three-layer structure in which the hypoechoic diaphragm fibrous tissue was located between the pleura and the peritoneum. Starting from the diaphragmatic crus at the mid-axillary line, the probe was gradually moving upward to the diaphragm apex, and the M-mode was used to record the change in thickness of the diaphragm while breathing after selecting the best image and exploration line, which could produce images that visualize the movement of the diaphragm over time and allowed accurate measurement of diaphragm thickness over a respiratory cycle (as shown in Figure 2) (14, 15). Diaphragm thickening fraction (DTF) was calculated following the equation below: DTF(%) = (Dtei−*Dtee*)/*Dtee*×100% (Dtei: diaphragm thickness at end-inspiration, Dtee: diaphragm thickness at end-expiration) (10, 16). The mean values of the DTF for each patient were calculated in three breathe cycles.

All the diaphragm ultrasound examinations were performed by a professionally trained physician, and the reproducibility was reported in Ref. (17).

## Data Collection

In this study, demographics, such as sex, age, underlying diseases, duration of mechanical ventilation, hospitalization time, and the time interval between diaphragm ultrasound and SBT implementation were recorded. Arterial blood gas was performed and recorded 0.5–1 h before performing the SBT. The right diaphragmatic excursion (DE) and speed, bilateral diaphragm thickness and DTF, the maximum and minimum diaphragm thickness and DTF were also recorded.

## Statistical Methods

The SPSS 25.0 statistical analysis software (IBM, Armonk, NY, United States) was used for data analysis. Count data were denoted as *N*, and chi-square test was used for group comparison. Normally distributed, continuous variables were denoted as $\bar{\text{x}}$ ± SD and non-paired *t*-test was used for intergroup comparison. Non-normally distributed, continuous variables were shown as [M (P_25_, P_75_)] in median (interquartile), and Wilcoxon’s rank sum test was used for intergroup comparison. For the indices measured in this study, the receiver operating characteristic analysis (ROC) was employed to assess the value of each index in predicting weaning outcome. The Youden index was used to determine the optimal threshold and its sensitivity and specificity. A *p-*value < 0.05 was considered statistically significant for the difference.

## Results

### Characteristics of Enrolled Patients

**FIGURE 3:**
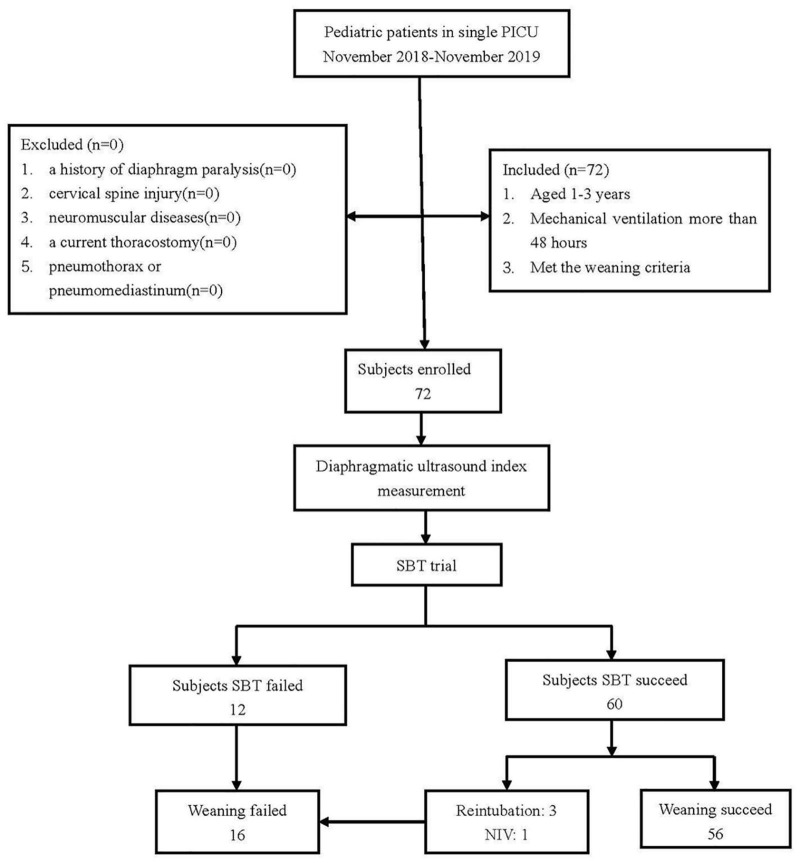
A flow chart of subject enrollment. SBT, spontaneous breathing trial; NIV, non-invasive ventilation.

As shown in Figure 3, a total of 72 patients were ready to undergo SBT in this study, and their primary diseases are displayed in Table 1. Sixty-four out of 72 patients were male subjects, and only 8 were female. Among them, 60 subjects passed a single SBT trial, and 12 subjects failed the SBT. Three of the patients who passed the initial SBT were reintubated, and another one patient was given non-invasive ventilation within 48 h after extubation. So, 56 subjects finally passed the weaning process (in the success group), and 16 subjects failed the weaning process (in the failure group). When compared between the two groups, the weaning success group and the weaning failure group, there were no significant differences in gender, age, partial arterial blood gas results (PH, PaO_2_, and PaCO_2_), and time intervals of ultrasound and SBT. The weaning success group had shorter days of mechanical ventilation and hospitalization than that in the weaning failure group (Table 2).

**TABLE 1:** General information of the patients.

Variables	Number
Cases	72
Male/female	64/8
Brain tumor	24
Virus encephalitis	4
Purulent meningitis	4
Autoimmune encephalitis	2
Convulsion	11
Pyemia	6
Pneumonia	6
Tracheal dysplasia	8
Congenital heart disease	6
Vehicle trauma	1

**TABLE 2:** Comparison of the parameters before weaning between the failure group and success group.

Parameters	Weaning failure group (*n* = 16)	Weaning success group (*n* = 56)	*p*-Values
**General information**			
Gender (male/female, *n*)	14/2	50/6	0.84
Age (months)	22.8 ± 6.8	23.7 ± 6.2	0.60
PRISM 3 score on PICU admission	10.0 (6.0, 13.5)	8.0 (4.0, 11.0)	0.14
**Clinical characteristics**			
pH	7.40 ± 0.08	7.41 ± 0.05	0.40
PaCO_2_ (mmHg)	42.5 ± 9.4	39.9 ± 4.9	0.30
PaO_2_ (mmHg)	91.8 ± 4.0	93.7 ± 3.6	0.07
Days of mechanical ventilation			
[d, M (P_25_, P_75_)]	9 (4, 14)	6 (5, 8)	0.05
Days of hospitalization			
[d, M (P_25_, P_75_)]	17 (14, 28)	9 (7, 11)	0.00
Time interval of ultrasound and SBT (h)	5.56 ± 2.25	5.46 ± 1.57	0.84
**Ultrasound results**			
Excursion (mm)	6.93 ± 2.77	9.36 ± 3.34	0.01
Speed (mm/s)	13.3 ± 8.7	14.6 ± 4.6	0.56
Dtee^R^ (mm)	1.13 ± 0.26	1.24 ± 0.29	0.17
Dtei^R^ (mm)	1.30 ± 0.31	1.57 ± 0.43	0.02
DTF^R^ (%)	15.9 ± 10.0	26.8 ± 16.7	0.02
Dtee^L^ (mm)	1.07 ± 0.27	1.20 ± 0.31	0.13
Dtei^L^ (mm)	1.22 ± 0.29	1.49 ± 0.40	0.02
DTF^L^ (%)	15.0 ± 7.7	24.3 ± 13.8	0.00
Dtee ^MIN^ (mm)	0.97 ± 0.36	1.12 ± 0.24	0.05
Dtee ^MAX^ (mm)	1.11 ± 0.41	1.32 ± 0.31	0.03
Dtei^MIN^ (mm)	1.19 ± 0.29	1.40 ± 0.35	0.03
Dtei^MAX^ (mm)	1.30 ± 0.35	1.66 ± 0.45	0.00
DTF^MIN^ (%)	10.0 ± 6.2	18.5 ± 9.6	0.00
DTF^MAX^ (%)	18.3 ± 9.2	32.7 ± 17.4	0.00

*Results are presented as median (IQR), $\bar{\text{x}}$ ± SD, or number.*

*Dtee^R^, right diaphragm thickness at the end of expiration; Dtee^L^, left diaphragm thickness at the end of expiration; Dtee^MIN^, minimum diaphragm thickness at the end of expiration; Dtee^MAX^, maximum diaphragm thickness at the end of expiration; Dtei^R^, right diaphragm thickness at the end of inspiration; Dtei^L^, left diaphragm thickness at the end of inspiration; Dtei^MIN^, minimum diaphragm thickness at the end of inspiration; Dtei^MAX^, maximum diaphragm thickness at the end of inspiration; DTF^R^, right diaphragm thickening fraction; DTF^L^, left diaphragm thickening fraction; DTF^MIN^, minimum diaphragm thickening fraction; DTF^MAX^, maximum diaphragm thickening fraction; PICU, pediatric intensive care unit; SBT, spontaneous breathing trial.*

### Diaphragm Ultrasound Measurements (the Right Diaphragm Excursion and Speed, the Bilateral Diaphragm Thickness, and Diaphragm Thickening Fraction)

As shown in Table 2, the right side of DE in the weaning success group was higher than that in the weaning failure group [(6.93 ± 2.77) mm vs. (9.36 ± 3.34) mm, *p* < 0.05]. However, there were no significant differences in the right side of speed between the two groups. For the diaphragm thickness, the end-inspiratory thickness of the diaphragm was significantly higher in the weaning success group than that in the weaning failure group under various conditions [right Dtei (Dtei^R^): (1.30 ± 0.31) mm vs. (1.57 ± 0.43) mm; left Dtei (Dtei^L^): (1.22 ± 0.29) mm vs. (1.49 ± 0.40) mm; the minimum Dtei (Dtei^MIN^): (1.19 ± 0.29) mm vs. (1.40 ± 0.35) mm; the maximum Dtei (Dtei^MAX^): (1.30 ± 0.35) mm vs. (1.66 ± 0.45) mm; all *p* < 0.05]. For the end-expiratory thickness of the diaphragm, the maximum thickness was higher in the weaning success group than that in the weaning failure group [(Dtee^MAX^): (1.11 ± 0.41) mm vs. (1.32 ± 0.31) mm, *p* < 0.05]. The DTF measured under various circumstances was also higher in the weaning success group than that in the weaning failure group [the right DTF (DTF^R^): (15.9 ± 10.0)% vs. (26.8 ± 16.7)%; the left DTF (DTF^L^): (15.0 ± 7.7)% vs. (24.3 ± 13.9)%; the minimum DTF (DTF^MIN^): (10.0 ± 6.2)% vs. (18.5 ± 9.6)%, the maximum DTF (DTF^MAX^): (18.3 ± 9.2) % vs. (32.7 ± 17.4)%, all *p* < 0.05].

### The Predictive Value of Each Index in the Weaning Success

**FIGURE 4:**
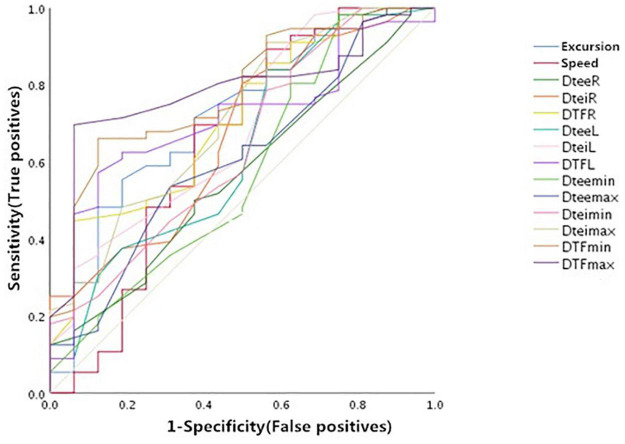
Receiver operator characteristic (ROCs) of the diaphragm ultrasound indices predicting weaning outcome before weaning. Dtee^R^, right diaphragm thickness at the end of expiration; Dtei^R^, right diaphragm thickness at the end of inspiration; DTF^R^, right diaphragm thickening fraction; Dtee^L^, left diaphragm thickness at the end of expiration; Dtei^L^, left diaphragm thickness at the end of inspiration; DTF^L^, left diaphragm thickening fraction; Dtee^MIN^, minimum diaphragm thickness at the end of expiration; Dtee^MAX^, maximum diaphragm thickness at the end of expiration; Dtei^MIN^, minimum diaphragm thickness at the end of inspiration; Dtei^MAX^, maximum diaphragm thickness at the end of inspiration; DTF^MIN^, minimum diaphragm thickening fraction; DTF^MAX^, maximum diaphragm thickening fraction.

As shown in Figure 4 and Table 3, the right DE, the maximum diaphragmatic thickness at end inspiration (Dtei^MAX^), and the DTF measured under various circumstances had moderate values of prediction for weaning outcome. The area under the ROC curves (AUC) (95% confidence interval) and the optimal threshold of the above indicators were as follows: DE (mm): 0.72 (0.58, 0.89) and 8.08 mm; Dtei^MAX^ (mm): 0.71 (0.57, 0.86) and 1.24 mm; DTF^R^ (%): 0.71 (0.57, 0.85) and 26.1%; DTF^L^ (%): 0.71 (0.58, 0.84) and 20.7%; DTF^MIN^ (%), 0.78 (0.66, 0.91) and 14.8%; DTF^MAX^ (%): 0.79 (0.67, 0.90) and 26.1%.

**TABLE 3:** Prediction efficiency of each diaphragm ultrasound index to the weaning outcome.

Parameters	AUC (95% CI)	*p*-Value	Threshold	Sensitivity (%)	Specificity (%)	Positive predictive value (%)	Negative predictive value (%)
Excursion (mm)	0.72 (0.58, 0.89)	0.01	8.08	55.4	81.3	91.2	34.2
Speed (mm/s)	0.65 (0.47, 0.72)	0.07	10.0	89.3	43.8	84.8	53.9
Dtee^R^ (mm)	0.57 (0.41, 0.72)	0.42	1.17	50.0	62.5	82.4	26.3
Dtei^R^ (mm)	0.67 (0.52, 0.82)	0.04	1.28	80.4	50.0	84.9	42.1
DTF^R^ (%)	0.71 (0.57, 0.85)	0.01	26.1	44.6	93.8	96.2	32.6
Dtee^L^ (mm)	0.63 (0.47, 0.80)	0.11	0.96	83.9	43.8	83.9	43.8
Dtei^L^ (mm)	0.69 (0.53, 0.84)	0.03	1.05	98.2	31.3	83.3	83.3
DTF^L^ (%)	0.71 (0.58, 0.84)	0.01	20.7	57.1	87.5	94.1	36.8
Dtee^MIN^ (mm)	0.58 (0.40, 0.75)	0.36	0.83	98.2	25	82.1	80.0
Dtee^MAX^ (mm)	0.61 (0.46, 0.77)	0.18	1.25	53.6	68.8	85.7	29.7
Dtei^MIN^ (mm)	0.64 (0.48, 0.80)	0.09	1.18	78.6	43.8	83	36.8
Dtei^MAX^ (mm)	0.71 (0.57, 0.86)	0.01	1.24	91.1	43.8	85	58.3
DTF^MIN^ (%)	0.78 (0.66, 0.91)	0	14.8	66.1	87.5	94.9	42.4
DTF^MAX^ (%)	0.79 (0.67, 0.90)	0	26.1	69.6	93.8	97.5	46.9

*Dtee^R^, right diaphragm thickness at the end of expiration; Dtee^L^, left diaphragm thickness at the end of expiration; Dtee^MIN^, minimum diaphragm thickness at the end of expiration; Dtee^MAX^, maximum diaphragm thickness at the end of expiration; Dtei^R^, right diaphragm thickness at the end of inspiration; Dtei^L^, left diaphragm thickness at the end of inspiration; Dtei^MIN^, minimum diaphragm thickness at the end of inspiration; Dtei^MAX^, maximum diaphragm thickness at the end of inspiration; DTF^R^, right diaphragm thickening fraction; DTF^L^, left diaphragm thickening fraction; DTF^MIN^, minimum diaphragm thickening fraction; DTF^MAX^, maximum diaphragm thickening fraction.*

## Discussion

In this study, we found that there were significant differences in the right diaphragm excursion (DE), the end-inspiratory thickness of the diaphragm (Dtei), and diaphragm thickening fraction (DTF) parameters between the weaning success group and the failure group when measured before the SBT trial. The indices, such as the right DE, the maximum diaphragmatic thickness at end inspiration (Dtei^MAX^), and the DTF, had moderate values of prediction for weaning outcome. These phenomena corroborated other investigations on the role of diaphragm ultrasound in the weaning policy of children on the mechanical ventilation (18, 19).

An estimated 20% of mechanically ventilated patients have difficulty in weaning from the ventilators, and the weaning process accounts for 40% of the whole ventilation time (20). In our study, the weaning failure rate was 22.0% (16/72), which was slightly higher than those in other articles. This variation might be due to differences in the definition of weaning failure, the types of underlying diseases, and the population studied. For this high rate of weaning failure, reliable tools are urgently needed to estimate the weaning outcome. The commonly used methods are as follows: (1) RSBI is the measurement of the ratio of respiration rate (RR, times/min) and tidal volume (VT, L) in the SBT, which is now widely used in clinical practice. The advantage of RSBI is that no cooperation is needed to perform, and it is applicable to children’s mechanical ventilation (21). Nevertheless, due to the different respiratory frequencies of children in different ages and the tidal volume related to weight, there is no fixed value of children’s RSBI threshold for reference. (2) Maximal inspiratory pressure (MIP) is the performance of a forceful inspiration against a closed airway so as to generate at least 1 s of maximal inspiratory pressure from the time of residual volume to the time of maximum inspiration. It reflects the effort level of the patient’s inspiration and evaluates the presence or absence of inspiratory muscle atrophy. The pressure generated by MIP was collectively induced by the elasticity of all inspiratory muscles, lungs, and thorax. The measurement of MIP in the ICU possesses the characteristics of safe and convenience. In adult studies, MIP > −20 cmH_2_O portends failure of weaning and MIP < −30 cmH_2_O portends success of extubation (22). It has a relatively high sensitivity (86–100%) and poor specificity (7–69%) (23). For children, the patients should be cooperative in performing inhalation, and it is hard to achieve for young children. (3) Airway occlusion pressure (P_0.1_) refers to the instantaneous airway pressure driven by nerve muscles 0.1 s after inhalation. As physiological respiration has little effect on this index and is generated through the contraction of the diaphragm, this index can be widely used in the assessment of the function of the respiratory center. Both higher and lower P_0.1_ have predictive significance for the failure of extubation. When P_0.1_ is high, it indicates the enhancement of the central inhalation drive; when P_0.1_ is low, it reflects the decrease in the central exhalation drive (24). However, the clinical value of P_0.1_ in children is seldom reported, making the prediction of weaning from the ventilator uncertain. In this study, we utilized the diaphragm ultrasound to evaluate the weaning outcome. The results showed that the parameters, such as the right DE, the maximum diaphragmatic thickness at end inspiration (Dtei^MAX^), and the DTF measured under various circumstances had moderate values of prediction for weaning outcome. Also, the bedside ultrasound used in this study was non-invasive, radiation free, economical, convenient, and not requiring special cooperation of patients.

In the children that received invasive mechanical ventilation, skeletal muscles wasting, such as in the diaphragm, was common and rapid. On the average of 6 days of mechanical ventilation, diaphragm thickness decreases by 11.1%, quadriceps femoris muscles thickness decreases by 8.62%, and continuous electrical impedance tomography shows an increase in the percentage of muscle fat, indicating a decrease in the muscle “quality” (18). In the pressure-controlled ventilation mode, the thickness of the diaphragm has a tendency to decrease, and there is an increasing trend before pressure support ventilation and extubation (8). Therefore, early initiation of spontaneous breathing, early weaning from mechanical ventilator, and alleviation of acquired diaphragmatic atrophy is the common goal of the intensive care unit and the rehabilitation department.

The analysis of Caifeng and colleagues found that the best prediction threshold for DE in adults was between 1.00 and 1.50 cm and DTF between 20.0 and 36.0%; the AUC of DE was 0.86, sensitivity was 0.79, specificity was 0.70, and the diagnostic odds ratio (DOR) was 10.6; The AUC of DTF was 0.84, sensitivity was 0.90, specificity was 0.80, and DOR was 32.5 (6). In this study, the DE of children was lower than that of adults: the optimal threshold was 8.08 mm, the AUC of DE was 0.72, the sensitivity was 0.55, and the specificity was 0.81. The DTF threshold of children was 26.14% on the right side and 20.71% on the left side, which were similar to that of adults. The study of Jung and colleagues showed that there were significant differences in DE and DTF between the adult weaning success group and the failure group, and the accuracy of DE prediction was higher, which was similar to this study (15). Due to the possible poor function of the lateral diaphragm and the issue of contralateral compensation, our study gave the minimum and maximum values of DTF on both sides and analyzed the extreme values on both sides; the predictive values were higher when DTF^MIN^ was 14.8%, DTF^MAX^ was 26.1%, and Dtei^MAX^ was 1.24 mm. Lee E-P and co-workers also found that the DTF was significantly different between the successful and failed extubation groups after extubation in children, and a DTF value of <17.0% was associated with extubation failure (18). IJland MM and coworkers found that 91.2% of the children were successfully extubated with a median DTF of 15.2%, which was lower than that in our study (25). The difference might be due to the difference in age and ethnicity. For pediatric diaphragm DE and thickness, they were positively correlated with the weight, which indicated that children with different weights needed corresponding prediction thresholds. As the predictive index for children weaning, DTF might be used as a relatively fixed index, which was more feasible than DE, and could avoid the impact resulting from weight changes along with growth and development (26).

There were some limitations in this study. First, due to the influence of flatulence on the left side, the measurement of DE on the left side, DE of the diaphragm ultrasound was limited. It was found that the right side of DE represented the overall DE in most studies. In order to minimize the bias of unilateral paralysis of diaphragm, this study included children who had not undergone surgeries of the thoracoabdominal approach. Second, the time interval between ultrasound and SBT was more than 30 min, and an SBT could only be carried out in approximately 5 h on average due to the influence of clinical work, which might affect the predictive value of diaphragm ultrasound. Third, although the results were statistically significant in this research, weaning from the mechanical ventilation was a process relying on the physicians’ overall assessment of the patients, which were independent of the authors. It should be prudent to apply the implementation of research findings into clinical practice. Last, the optimal prediction threshold of DE or the sensitivity of DTF in this study was lower, and the specificity is similar to that of adults. Although cutoff values were calculated statistically in the study, there were too many overlapping values. Using these breakpoints individually at the bedside should be done with caution. Also, as a single center study, the numbers of subjects and disease types were limited, and multicenter studies with larger sample sizes were needed for confirming the predictive value of diaphragmatic ultrasound in weaning from mechanical ventilation in children.

In conclusion, diaphragmatic ultrasound is feasible in guiding children weaning from mechanical ventilators in clinical practice. The right DE, DTF, and Dtei^MAX^ indices had moderate values of prediction for weaning outcome. Among them, DTF might be able to avoid the impact caused by the diaphragm development, acting as the more reliable predictive index for weaning in children.

## Data Availability Statement

The original contributions presented in the study are included in the article/supplementary material, further inquiries can be directed to the corresponding authors.

## Ethics Statement

The studies involving human participants were reviewed and approved by the Scientific and Ethics Committees of Children’s Hospital of Fudan University. Written informed consent to participate in this study was provided by the participants’ legal guardian/next of kin.

## Author Contributions

YY and LH together completed the data collection, statistical analysis, and manuscript writing. JT and SW conceptualized the study, designed the study, interpreted the data, and revised the manuscript. WC and GL provided administrative support, patients, and data interpretation. HZ participated in the data collection and assembly. All authors contributed to the article and approved the submitted version.

## Conflict of Interest

The authors declare that the research was conducted in the absence of any commercial or financial relationships that could be construed as a potential conflict of interest.

## Publisher’s Note

All claims expressed in this article are solely those of the authors and do not necessarily represent those of their affiliated organizations, or those of the publisher, the editors and the reviewers. Any product that may be evaluated in this article, or claim that may be made by its manufacturer, is not guaranteed or endorsed by the publisher.
